# Lived Experiences of Older Adults Using Wearables With Real-Time Feedback: Phenomenological Study

**DOI:** 10.2196/71509

**Published:** 2026-04-29

**Authors:** Tien-Ying Lu, Aurora Rosato, Seraina Anne Dual, Sanna Kuoppamäki

**Affiliations:** 1 Department of Biomedical Engineering and Health Systems KTH Royal Institute of Technology Stockholm Sweden

**Keywords:** wearables, physical activity, multimodal feedback, synchronization, older adults, interview study

## Abstract

**Background:**

Wearable devices with real-time feedback (WRFs) provide increasing opportunities to enhance physical activity and improve rehabilitation through collecting and processing health-related data. Real-time feedback (RTF) from the device is expected to result in a more dynamic, coordinated, and synchronous rhythmic activity, defined as step-by-step movements mediated by the real-time heart rate feedback. However, age-specific characteristics in the user engagement with WRFs integrating real-time audio feedback have largely remained unexplored.

**Objective:**

This study explores the lived experiences of older adults using wearables with RTF to uncover motivations, aspirations, and hindering factors in their engagement with WRFs in rhythmic activity. The study explores narratives that older adults articulate in their previous use of wearables for physical activity, their experiences with WRFs during rhythmic activity, and their meaning-making of the interactive features enhancing the synchronization of the movement during rhythmic activity.

**Methods:**

The study was conducted as a qualitative interview study with 18 older adults who used a WRF for rhythmic activity during a 3-week period in their home environment. The wearable used in the study is a chest-band sensor device that helps users to synchronize their steps with their heartbeat through the provision of real-time audio feedback. The material consists of semistructured interviews before and after using the device. Material from the semistructured interviews was analyzed with an interpretative phenomenological analysis method.

**Results:**

The study identified four main themes characterizing older adults’ lived experiences with wearables, which are (1) use of wearable technologies without RTF in daily life, (2) embodied rhythmic negotiation with RTF, (3) interpretation of health data with RTF, and (4) temporal trajectories of device engagement with RTF. Older adults demonstrated intentional distancing from wearable technologies rather than simple disuse, prioritizing authentic bodily experiences over external validation. Their engagement was fundamentally relational, mediated through trusted social networks, and required dialogical support for data interpretation. Device-guided movement synchronization created contextually situated challenges that varied significantly based on environmental demands, individual bodily capacity, and exercise routines. Extended temporal engagement transformed participants’ relationships with the technology from initial disruption to potential integration, with RTF serving as a bridge toward enhanced embodied awareness when carefully designed.

**Conclusions:**

The study concludes that RTF from the device can enhance synchronization and bodily awareness, but meaningful engagement requires adaptive designs that respect older adults’ authentic movement practices, accommodate their relational approach to technology validation, and allow sufficient time for embodied competency development.

## Introduction

### Background

Physical activity (PA) is an important predictor for active and healthy aging; yet, few older adults meet the recommended levels of PA [1]. Wearable devices with real-time feedback (WRFs) having sensors provide increasing opportunities to enhance PA through collecting and processing health-related data [2,3]. Despite their potential, the long-term user engagement with wearables remains a challenge [4-6]. In particular, age-specific characteristics in the user engagement with interactive wearables with real-time feedback (RTF) have remained largely unexplored.

This study explores the use of wearables with and without RTF to uncover the lived experiences of older adults in their engagement with interactive wearables. WRFs can be defined as body-worn technologies that implement technologies, such as sensors, to measure biomedical information, combined with modalities, such as haptic or auditory feedback, that help users to adjust the movement to improve the performance in PA [7]. Rhythmic activity can be defined as a form of PA that incorporates elements such as auditory feedback, music, and social interaction, which involve coordinated movements synchronized with music [8-10]. In this study, we define rhythmic activity as a step-by-step movement (eg, walking or running) that is mediated by a device-guided synchronization of step and heart rate.

Older adults, defined as an age group of 65 years and older, are considered an important user group of WRFs due to the potential of wearables to improve PA, rehabilitation, and cardiovascular health [1,11-14]. Due to demographic changes accompanied by population aging, wearables as digital health interventions are expected to contribute to health promotion and prevention of disease among this demographic [15]. However, the adherence with these interventions still remains a challenge [4-6]. Older adults’ adherence with wearables is mediated by their health, motivations for PA, and digital skill literacy, which can impact the use of wearables that require dexterity, mobility, or vision [16,17]. Older adults emphasize enjoyment of social interaction as a motivation to be physically active [1], and wearables can enhance this through the provision of visual or auditory feedback [12]. The perceived appeal of visual or auditory feedback, however, is individual and mediated by the lived experiences in later life. This suggests that affordances embedded in wearables are not directed only toward improving performance, but also delivering a sense of self-awareness [13]. Considering the individual differences in older adults’ motivations, it becomes important to recognize their lived experiences when exploring their adherence to WRFs.

The aim of this study is to explore the lived experiences of older adults using wearables with RTF in rhythmic activity. The study explores narratives that older adults articulate in their previous use of wearables for PA, their experiences with WRFs during rhythmic activity, and their meaning-making of the interactive features enhancing the synchronization of the movement during rhythmic activity.

### Wearables With RTF

Wearable technologies are designed and used for enhancing PA in health promotion, preventative health, and treatment of health conditions [18]. As *self-care technologies*, wearables allow the user to take responsibility for managing their own health independently, without external intervention from the health care provider [15,19,20]. As a *medical technology*, wearables can be used before medical diagnosis to monitor PA levels, and detect and predict the onset of clinical conditions [21].

Wearable technologies include a variety of applications, such as smart watches and chest bands, and these technologies integrate features, such as instructions, self-monitoring, and reminders, along with customizable options [22,23]. These interactive features, such as goal setting, feedback provision, and motivation tracking, are increasingly implemented in day-to-day applications [23]. RTF refers to an interaction mechanism that provides haptic, auditory, or visual feedback to the user simultaneously with the user’s movement, instead of after the movement or activity has ended [2,3]. Machine learning techniques have enabled accurate and rapid human gesture recognition, providing RTF to users [24]. WRFs have been investigated among athletes in the prevention of injuries, improvements of performance [2,25], as well as in rehabilitation [26]. For older adults, RTF has been shown as a preferred feature in wearables [11]. In comparison to other interactive features, RTF, as a rather novel feature, has nevertheless been less implemented in commercially available wearables [2].

RTF enables reactions from the user while the action or movement is ongoing. Auditory feedback allows users to be observant of the environment and fits into more dynamic settings compared with both visual and tactile guidance [27]. Through sound interaction, users can coordinate and stabilize movements with internal or external imposed rhythms [28]. Auditory feedback can be used in a variety of physical activities, ranging from guiding speed to correct movements [25], supporting coupling steps and breathing in day-to-day running exercises [27], to guiding static breathing exercises [29]. Beat-by-beat stepping to the heart rate has shown improved heart function in athletes; however, without assessing motivational aspects of this interaction [30]. As an alternative to the auditory cues, the use of music can increase motivation and engagement in cardiovascular rehabilitation [31].

### Factors Influencing Adherence With Wearables Among Older Adults

Wearables are accessible and easy to use, but the adherence, defined as long-term user acceptance and engagement with the device, remains a challenge [4-6]. The ownership of wearables is defined by sociodemographic factors, such as age and gender. Younger groups with higher levels of education and full-time employment are more likely to own a wearable compared with other demographic groups [32]. Older adults’ motivations for using a wearable may also significantly differ from other age cohorts regarding the perceived value and ease-of-use of the device [11,14,33-35].

Long-term adherence to wearables is determined by a combination of individual motivation, ease-of-use, and perceived value of the wearable, which subsequently predicts whether or not the device will be integrated into the user’s life [11,33]. Older adults must be motivated by the usefulness of the device and its added value to PA [11,36]. Formal and informal support and guidance in having access to the device and continuing its use are connected to long-term adoption [37]. These individual factors are expected to differ between older adults, resulting in heterogeneous use of wearables depending on various individual and social determinants [10,16,17].

Emotional, sensory, and social influences also impact the user engagement with wearables [13,38,39]. Long-term attachment with wearables requires that these devices elicit positive emotional responses among users [40]. When wearables are framed as an assistive technology that aims to compensate for age-related functional or cognitive losses, older adults might abandon these devices due to (negative) user images [36]. Wearable devices can be perceived as motivating or condescending depending on how freely older adults can make autonomous choices about PA [34].

Accordingly, older adults face barriers to wearable adoption, including issues about usability, affordability, and accessibility, which can lead to social isolation and marginalization from digital health innovations [41]. At the same time, digital technologies offer significant opportunities for older adults, who often have more leisure time and are financially independent, to seek health-related information and maintain self-care [15,42]. Fewer phenomenological studies are focused on exploring older adults’ experiences with rather novel wearable technologies in their home environments. This methodological approach allows us to capture the essence of subjective experiences and the perceived value they derive from using wearables through first-person descriptions [41-44] and the meanings they attribute to the interactions with wearables [15,45]. The research questions (RQs) are the following:

- RQ1: What kind of narratives do older adults articulate in the use of wearables for PA?
- RQ2: How do older adults experience the use of wearables with RTF in rhythmic activity?
- RQ3: What kind of meanings do older adults associate with the interactive features of a WRF during rhythmic activity?

## Methods

### Study Procedure

**Figure 1:**
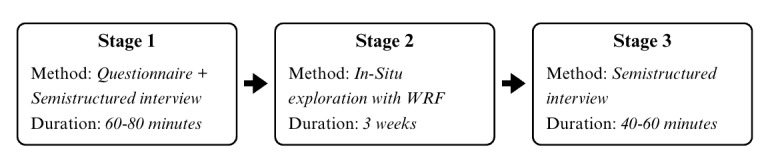
Method and duration for each study stage.

This study consisted of three stages: (1) semistructured interview and a questionnaire before using WRF, (2) a 3-week use of WRF for PA (referred to as in-situ study), and (3) a semistructured interview after using WRF. Figure 1 presents the method and duration of each stage.

In the first stage, we conducted a semistructured interview for participants to investigate their motivation for PA, self-perceived health and well-being, previous experience with technologies, and previous interactions with wearables for PA. Participants were also given a questionnaire measuring their PA level, self-perceived health, and sociodemographic characteristics.

In the second stage, participants were asked to use a WRF for a 3-week period in their home environment. They were asked to use the device at least 3 times per week during their PA routines, including any rhythmic activity, such as walking, running, or jogging. Each participant was shown how to use the device, and the device was set up and connected to their mobile phones. They also received detailed written instructions for using the device. A 3-week period for in-situ study was selected to ensure the feasibility of the procedure as a common period in digital health intervention studies [46].

In the third stage, we conducted semi-interviews to explore participants’ lived experiences with the device, focusing on their self-perceived perceptions of the device and its impact on motivation and adherence with rhythmic activity.

### Recruitment of Participants

Participants in this study were recruited through convenience sampling at the Swedish School of Sport and Health Sciences. An open invitation was distributed to those who attended the physical exercise sessions 3 times a week at the Swedish School of Sport and Health Sciences. Since the WRF device was originally designed for athletes to improve their performance, we recruited physically active older adults to ensure that the device was considered meaningful and safe for the user group to allow a phenomenological approach to their experiences [47,48]. Other inclusion criteria were (1) participants aged older than 65 years, (2) able to exercise independently without assistive devices, (3) own a mobile phone that can download the app and could carry the phone during PA, (4) able to adequately see and hear (with or without a hearing aid) the wearable devices’ RTF, and (5) able to read and speak English to follow the WRF’s instructions.

In total, 21 older adults were recruited, and 18 of them completed all 3 stages in the study. The sample size was determined based on the principles of qualitative research and purposive sampling, where having 20 participants is often considered a saturation point after which little or no new information is generated from 1 participant group [49].

### Data collection

#### Questionnaires

At the beginning of this study, a questionnaire designed and based on guidelines from the Swedish National Board of Health and Welfare [50,51] was distributed for older adults to subjectively assess their health status and PA (Multimedia Appendix 1). Weekly PA time with multiple-choice questions for both high- and low-intensity activities was reported. Self-perceived health is measured using a 5-point scale covering self-perceived physical health. Other information includes age, gender, marital and family status, living arrangements, and finally, a section for additional comments. In this study, the questionnaire results were used as background information on the participants’ health and PA.

#### Semistructured Interview

We conducted 2 rounds of semistructured interviews with the participants before and after they used a WRF for 3 weeks. All interviews, lasting approximately 45-60 minutes, were audio-recorded and later transcribed for analysis. Interviews were conducted by 2 researchers, and they were collected as individual in-person interviews. Interview guidelines were prepared for both interviews to ensure that the procedure was similar in all interviews (Multimedia Appendices 2 and 3).

In the first round of interviews, participants were asked about their life situations and daily routines in PA, including questions about their PA patterns, motivations, challenges, and perceived health. Questions were asked about their previous use of digital health technologies and wearables. For those who had used wearables previously, we asked about the contexts, benefits, and challenges of wearables use, while nonusers of wearables were asked about their expectations and willingness to adopt such technologies. This interview also touched upon the social aspects of wearables for PA, including preferences for group exercise and peer advice on technology adoption. In this round of interviews, we gathered comprehensive qualitative data about participants’ relationships with both PA and technology (Multimedia Appendix 2).

After participants had used the WRF for 3 weeks, they attended the second round of interviews. Participants were asked about their experiences, perceptions, and attitudes toward the device based on their usage of it in rhythmic activity (walking, jogging, or running). Participants were encouraged to reflect in detail on their interactions with the device, including possible challenges. Questions relating to user experience sought to understand participants’ motivations and emotional responses toward WRF and capture the subjective, contextualized characteristics of their engagement. We probed participants to evaluate the RTF and whether or not it was seen as supportive. Usability-focused questions assessed the ease of use and technical barriers. Finally, we addressed acceptance and future use of WRFs to inspect their openness to long-term adoption for more advanced interactions, such as conversational features or personalized feedback mechanisms (Multimedia Appendix 3).

#### In-Situ Study of the Device

In-situ study is valuable for exploring health-related experiences because it honors participants’ situated knowledge and lived contexts [52,53]. Engaging with the embodied and sensory dimensions helps to reveal nuanced insights into how contextual factors shape health and well-being [54,55]. Moreover, this method allows participants to actively take control of their experiences and foster their agency [56,57]. This aligns with our intention to offer participants more flexibility and autonomy throughout the study.

**Figure 2:**
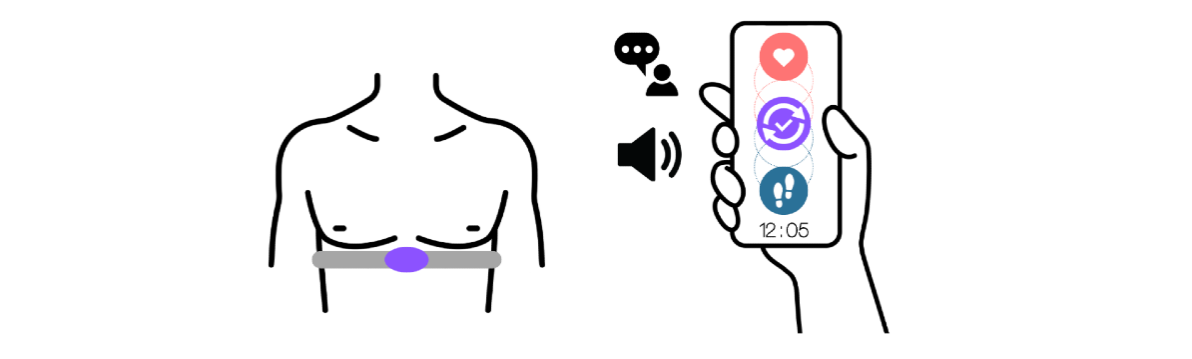
Schematic diagram of the WRF—it consists of a chest-worn heart rate and step rate sensors and a mobile app, which provides both real-time visual and auditory feedback. WRF: wearable device with real-time feedback.

The WRF used in this study, Counterpace (Pulson Inc), is a commercially available novel wearable that guides wearers to step precisely between heartbeats during rhythmic activity. Its chest-worn electrocardiography sensor detects the heart state (contract or relax), and an accelerometer detects the walking state (stepping). The connected mobile app provides visual information (step rate, heart rate, and synchronization time and percentage). It also provides voice instructions and metronome beats for wearers (Figure 2). By synchronizing step pacing with heartbeats, the device promotes a step-by-step movement that we define as *rhythmic activity* in this study. There are 2 main reasons why this device was chosen. First, this WRF is developed for rhythmic activity, which is expected to positively influence cardiovascular health [30], with possibly greater benefits for older adults due to increased vascular stiffness [58]. However, its usage and benefits for older adults remain underexplored. Second, although this WRF was originally developed for elite runners and high-performance training, its design has not considered accessibility or inclusivity.

By introducing this technology to older adults, we aim to understand whether its potential benefits could be motivating and meaningful for this population, and to uncover the specific challenges they encounter in using it. Their activity and heart rate were recorded by the mobile app and stored in an anonymized format on a cloud-based service. Participants were encouraged to contact the researchers for technical support throughout the study.

#### Participants

Of the 21 recruited participants, 18 completed all stages of the study. Table 1 presents their sociodemographic characteristics. The majority (11/18) were between the ages of 70-75 years, with 3 participants aged 66-67 years, and 1 participant older than 80 years, with a mean age of 72.1 years. Most participants were females (12/18). For the PA level, the majority (15/18) reported 90 minutes or more of high-intensity activity per week, with 2 participants reporting an intermediate level (30-89 minutes), and only 1 reported a low level (0-30 minutes). Most participants (10/18) also engaged in 90 minutes or more of low-intensity PA per week. Moreover, 6 participants reported high perceived physical health (scores 1-2), while 4 reported low perceived physical health (scores 5-6).

**Table 1:** Participants’ sociodemographic characteristics, including age, gender, physical activity level, and perceived health (n=18).

ID	Age (y)	Sex	High-intensity PA^a^ level (minutes per week)	Low-intensity PA level (minutes per week)	Perceived physical health^b^ (score 1-6)
P1	70	Female	≥120	30-89	High
P2	74	Male	90-119	30-89	Medium
P3	70	Female	90-119	30-89	High
P4	70	Male	≥120	90-119	Medium
P5	78	Female	≥120	≥120	Low
P6	72	Male	90-119	30-89	Medium
P7	76	Male	30-89	30-89	Medium
P8	71	Female	≥120	0-30	Medium
P9	82	Male	0-30	30-89	Medium
P10	70	Female	90-119	90-119	Medium
P11	70	Female	90-119	30-89	Medium
P12	72	Male	≥120	≥120	High
P13	78	Female	≥120	≥120	High
P14	72	Female	30-89	90-119	Medium
P15	67	Female	≥120	90-119	High
P16	66	Female	≥120	≥120	Low
P17	72	Female	≥120	90-119	Low
P18	67	Female	90-119	≥120	Low

^a^PA: physical activity.

^b^Perceived physical health was assessed by asking participants, “How satisfied are you with your health?” using a 6-point Likert scale, where 1 indicated very satisfied, and 6 indicated not satisfied at all. The responses were grouped as follows: high=scores 1-2, medium=scores 3-4, and low=scores 5-6.

### Analytical Approach

This study used a phenomenologically informed qualitative approach, drawing on the principles of interpretative phenomenological analysis (IPA) [47,59] to explore how older adults make sense of their experiences with wearable technology in PA. IPA is particularly suited to examining how individuals interpret, reflect on, and assign meaning to personally significant experiences, with an emphasis on embodiment, temporality, and the situated nature of meaning-making.

While the early stages of the analysis were guided by a thematic analysis conducted by 3 researchers to navigate a rather large and diverse dataset [60,61], the final interpretative process was anchored in phenomenological inquiry. The goal was not simply to identify recurring patterns but to understand participants’ lived experiences in depth, including how they perceived, emotionally responded to, and negotiated the role of wearable devices in their embodied PA routines.

The analysis followed a recursive and idiographic process, beginning with repeated readings of each interview transcript to immerse in the participants’ narratives. After preliminary notes were made, segments that revealed how participants made sense of their relationship with WRF were identified and grouped. These segments were later interpreted within the broader biographical, social, and emotional context, maintaining an interpretative stance that sought to balance empathic understanding with critical reflection, a process central to IPA’s double hermeneutic process. Although initial thematic groupings were constructed, these were reevaluated through a phenomenological lens to ensure that the final themes captured the depth, nuance, and individuality of participants’ meaning-making. Particular attention was paid to heterogeneity among the narratives articulated by older adults, not as demographic variance, but as diverse experiential positions shaped by bodily awareness, social scaffolding, emotional responses, and contextual life circumstances. The final themes represent not only recurring patterns but rich, interpretative accounts of how wearable technologies were encountered, negotiated, incorporated, or even resisted in older adults’ daily lives.

### Ethical Considerations

The research has been approved by the Swedish Ethical Review Authority (references 2023-00426-01 and 2024-06934-01). All participants gave informed verbal and written consent for participation in the study and for publication of anonymized extractions from the data. Before participants signed the informed consent, they were given both written and verbal information about the effects of adapting this device in their PA routines and potential risks in participating in the study. Participants were given the possibility to ask questions and withdraw from the study at any time. The privacy and confidentiality of participants were protected by giving all participants a pseudonym (numeric ID) that was used in the analysis and reporting of the results. Participants were given a small financial compensation of a gift card worth 500 SEK (US $54) after they completed their participation in the study. The amount of financial compensation was not specified in the recruitment material.

## Results

### Overview

The analysis revealed four superordinate themes: (1) use of wearable technologies without RTF in daily life, (2) embodied rhythmic negotiation with RTF, (3) interpretation of health data with RTF, and (4) temporal trajectories of device engagement with RTF (Table 2). The results capture the embodied, temporal, and situated nature of participants’ meaning-making processes, revealing how relationships with technology evolved. A detailed analysis table can be found in Multimedia Appendix 4. The findings demonstrate the depth and heterogeneity of lived experiences, showing how wearable adoption is deeply personal and contextual, shaped by participants’ ongoing negotiation between technological mediation and their intuitive, embodied understanding of PA.

**Table 2:** Themes from the interpretative phenomenological analysis.

Superordinate theme	Subordinate theme
Use of wearables without RTF^a^ in daily life	Embodied autonomy and resistance to digital mediation Contextual and temporal meaning-making Tensions and ambivalent emotional responses
Wearables with RTF: embodied rhythmic negotiation	Disrupted natural flow Learning and guided transformation Raising bodily awareness through real-time multisensory feedback
Wearables with RTF: interpretation of data	Relational meaning-making of health data Technological barriers hindering PA enhancement
Wearables with RTF: temporal trajectories of device engagement	Disruption and emerging connection in limited engagement Awareness and growth in moderate engagement From discipline to integration in extended engagement

^a^RTF: real-time feedback.

### Use of Wearables Without RTF in Daily Life

#### Overview

Participants expressed diverse narratives with wearable technologies, shaped by personal values, bodily autonomy, and lived experience. Many resisted using digital tools, such as tracking apps, prioritizing internal bodily awareness over external data. Engagement was also influenced by social networks and emotional responses, such as stress or pressure. Technology use reflected intentional choices rooted in identity, context, and trust.

#### Embodied Autonomy and Resistance to Digital Mediation

The lived experience of wearable engagement was characterized by participants’ assertion toward embodied autonomy over digital health mediation. This theme represents a profound stance where participants actively resisted and defended their authentic relationship with their bodies against what is perceived as technological intrusion. This theme emphasizes that participants’ adoption of wearables goes beyond simple preference, revealing a deeply held commitment to preserving their agency and embodied experience in health and wellness practices.

Most participants owned one or more wearables but deliberately remained disconnected from them, manifesting as “ownership without utilization,” where possession of the technology coexisted with intentional non-engagement. For instance, in P2’s statement, we see more than simple disuse; we see intentional distancing from technology.

I have a smartwatch, but I do not want to use it during physical activity. I keep it somewhere at home.

P2, male, 74 years

The way participants physically removed themselves from their devices reflects a conscious choice to make the technology irrelevant in their daily lives, expressing a more profound desire to protect their genuine, lived experience of the body and to maintain psychological boundaries against letting technology shape their bodily awareness.

The experience of external pressure from family, friends, or PA instructors to adopt wearables revealed another dimension of resistance. One participant’s steadfast position of remaining uninterested was evident as he was,

…uninterested in adopting wearables, even though my son uses a smartwatch and tries to persuade me to wear it too. I do not think it is that important to know how many steps (I took during my walks) because I already feel it roughly.

P4, male, 70 years

This illuminated how this resistance was not rooted in inaccessibility, but rather in a principled stance about the characteristics of health monitoring. This resistance is grounded in an understanding that external validation of bodily states has less value compared to their own physical experience.

For some participants, PA was experienced as unencumbered bodily engagement, where additional equipment was perceived as interference with pure movement. One participant articulated this essence with particular clarity:

For me, that is why I like jogging and running. I do not want... (to use wearables). It should be easy to train my body... It should be just as easy as possible to move around.

P10, female, 70 years

Her lived experience revealed how wearable technology was perceived as disrupting the unmediated nature of exercise. Participants’ preference for exercise as “easy” was not about avoiding difficulty, but about maintaining a natural and straightforward connection between mind and body during exercise, suggesting that participants viewed their rejection of wearables as a protective measure for something fundamental to their identity and well-being.

Moreover, the lived experience of health monitoring was characterized by participants’ profound trust in their own bodily awareness over technological feedback, revealing a sense of self-awareness grounded in direct physical experience rather than external measurement. One participant explained:

I have an Apple Watch, which I do not use much... I look at my heart rate because of the type of exercise I do. But I am kind of structured anyway.

P14, female, 72 years

Her experience illuminated how participants privileged their embodied and intuitive understanding of their bodies over external technological validation. Their narrative suggested that they possessed an internal system for understanding their physical needs and responses, rendering external monitoring redundant. This represented not only the rejection of health monitoring but also a commitment to internally generated, experientially based health awareness that participants trusted more than digital data.

#### Contextual and Temporal Meaning-Making

Participants’ engagement with wearables unfolded as profoundly contextual and temporal, where meaning and utility shifted across different life circumstances and personal preferences. Participants’ interactions with technology were not static, but a dynamic process of meaning-making that they continually reflected upon in relation to contextual needs and values. The temporal dimension of this engagement also suggests that relationships with wearable technology were biographically embedded within participants’ life stories and subject to reinterpretation over time.

Contextually, the technology preference emerges through participants’ definition of what “useful health monitoring” entails, revealing highly individualized criteria. One participant articulated:

(I found) a basic step-counter in the form of a phone-based application sufficient to track most basic physical activities.

P1, female, 70 years

Her use of the word “sufficient” was particularly intriguing, which shows that she had developed precise measurements for what constitutes adequate, allowing her to be free from external recommendations or marketing messages about optimal health tracking. This preference revealed how participants proactively set their own boundaries around technological complexity and determined what digital tools aligned with their technological sophistication, necessity, and manageability.

Additionally, selective engagement with personal data interpretation emerged as participants demonstrated discernment about which aspects of wearable technology resonated with their personal understanding of health and movement. One participant described his experience:

(During walking and running) It is fun to see how far you have gone, how fast you have gone, what kind of mileage you have got…

P4, male, 70 years

This illustrates how enjoyment emerged from specific data points that connected with his personal measurement of a meaningful activity. His emphasis on “fun” shows that the engagement was not just driven by arbitrary fitness imperatives but by his intrinsic interests in tracking and inquisitiveness in his performance. This selective engagement demonstrates how participants acted as curators of their technological experience by choosing to interact with elements that enhance their PA.

On the negative side, abandonment or pause of usage occurred due to temporal change in their relationships with wearables, disclosing how the purpose and utility of these devices were subject to changing life circumstances, which vary from the loss of family members, injury, chronic diseases, relocation, and so on. Another participant described how her usage of wearables shifted over time:

I used to have one (Garmin watch) many years ago when I was running. It was useful then, but now I do not use it anymore.

P13, female, 78 years

The phrase “*it was useful then*” implies that usefulness was not an inherent property of the technology, but rather a mixture of the device’s capabilities and the participant’s needs at that particular period in her life. Ultimately, the disengagement with wearable devices was the natural evolution over time, and could not be indicative of their usability or utility.

Finally, we discovered a technological curiosity among participants who have not tried any wearable devices, as evidenced by their openness to digital health tools, particularly those that offer well-structured guidance for PA. A participant expressed that,

I appreciate and I believe in technology. And I had tried several fitness apps previously…

P3, female, 70 years

This highlights a broader exploratory relationship with digital health resources. The experimental approach to “try and find” a new tool indicated that participants do not regard technology adoption as a firm commitment to 1 tool, thereby retaining control over their engagement. This sense of curiosity also reflects active meaning-making, as participants need various technologies and can identify those that resonate with them, rather than simply consuming them passively.

#### Tensions or Ambivalent Emotional Responses

The use of wearables among older adults was significantly influenced by their social and emotional aspects. This subtheme illuminated the social nature of technology adoption among this demographic, which was often embedded within and mediated through personal networks and communities. Similarly, their engagement was marked by complex, often conflicting feelings, demonstrating that wearables are not a neutral tool but an emotionally charged artefact that can have a profound impact on participants’ sense of self and their relationship with their bodies.

The social dimension of wearable adoption was notably influenced by participants’ trust toward others, who served as mediators of acceptance and attachment. One participant described how her wearable usage began through professional guidance:

I got to know about my smart ring from a health engineer. She is also very skilled in various aspects of health. And she was the one who suggested this ring.

P17, female, 72 years

P17’s emphasis on the health engineer’s expertise suggested that this referral carried weight in her decision-making because it came from individuals whose judgment she believed. The technology gained legitimacy through respected social connections, and at the same time, this mediation represented not simply an information transfer but also the validation of technological benefits.

Some participants also mentioned attending webinars and lectures on new technologies, suggesting that older adults’ technology adoption was facilitated through community-based learning environments where they could explore within supportive contexts. This furthermore shows that older adults’ engagement with wearables was fundamentally relational rather than individualistic. This social scaffolding appeared crucial for older adults who might otherwise feel intimidated by or skeptical of wearable technology, providing both practical guidance and emotional support for technology exploration.

Participants demonstrated remarkable emotional awareness in expressing these complex relationships with wearables. We found that accessing and tracking health data is a double-edged sword, raising paradoxical feelings. Participants, such as P6 (male, 72 years) and P15 (female, 67 years), reported feeling negative emotions, including stress, confusion, and anxiety, after noticing an unexpected decline in their PA levels. Their experience exemplifies how data can disrupt their perceived well-being. More explicitly, P15’s experience illuminated the tension between the potential benefits and its emotional costs:

I have a step calculator (on the phone). But I do not want to check it all the time. It is competitive and stressful... I think that is stressful.

P15, female, 67 years

P15’s experience revealed how the pressure of goal-oriented tracking conflicted with her authentic relationship to PA, leading her to limit her engagement. The repetition of “stressful” in her statement emphasized that constant monitoring could hinder her view of the technologies. Her description of tracking as “competitive” suggested that it is hard to disconnect the quantitative data from external standards, such as suggested steps per day, even though well-being should be very individual and personalized.

### Wearables With RTF: Embodied Rhythmic Negotiation

#### Overview

Participants experienced RTF as both disruptive and transformative. While initial use challenged authentic movement patterns, some developed new bodily awareness. Engagement was shaped by sensory feedback, usability, and the need for support in interpreting health data. The technology offered both opportunities for connection and barriers to meaningful use.

#### Disrupted Natural Flow

Participants’ experiences with WRF revealed a tension between guided synchronization and their authentic rhythm. To synchronize their heart rate and step rate, there was a disruption to their PA flow. Participants became conscious about how they move, and their exercise session became a deliberate practice. For participants, the device’s requirements created a confrontation with their bodily rhythms. As P8 (female, 71 years) reflected, the expectation to “*go with your heart*” triggered a realization about her relationship with her body:

I have realised that I have never followed my heart. I walk much faster.

P8, female, 71 years

This shows how the synchronization challenge exposed participants’ disconnection from their internal rhythms.

Moreover, a sense of inauthenticity and bodily disharmony prevailed when they attempted synchronization. Participants described feeling caught between complex demands, including their original moving patterns, environmental constraints, and the device’s rhythmic imperatives. All the demands combined led to a cognitive burden for participants, which requires constant attention and internal negotiation. Participants experienced this burden as fragmenting their usual unrestricted movement. As P18 (female, 67 years) described, “*even momentary lapses in attention resulted in failing synchronisation*,” revealing how the device demanded a sustained cognitive effort with bodily rhythm that felt foreign to them.

Participants also experienced synchronization widely across different movement contexts. Flat terrain emerged as providing the most conducive environment, while stairs and uneven surfaces created barriers to synchronization. This suggests that the experience of rhythmic alignment is deeply contextual, shaped by the interplay between bodily capacity, environmental demands, and technological requirements. Environmental factors thus intensified their cognitive burden. Icy conditions, uneven terrain, and slopes created more layers of complexity that further estranged participants from feeling natural. P13’s (female, 78 years) experience of “*when walking downhill, I need to slow down my steps to remain in sync*” illustrates how environmental contexts compounded the sense of imposition.

#### Learning and Guided Transformation

Despite cognitive struggles, some participants experienced a meaningful transformation in their relationship with synchronization. Their learning experience emerged as a gradual integration of guided rhythm in PA. P3 (female, 70 years) described the experience “*becoming like a dance*” once synchronized, suggesting a qualitative shift from effortful practice to embodied harmony. This transformation necessitated a reorientation in how participants perceived their bodies and movement. The warming-up process described by P3 also suggests that synchronization required not just physical preparation, but also an attentional preparation that allowed for the integration of heart rhythm awareness into movement consciousness.

The desire expressed by some participants (P12 and P14) to extend synchronization to other activities not included in the connected app, such as gym workouts or group settings, indicates an emerging appreciation for the device’s potential in various scenarios. However, they noted a lack of guidance for these contexts as problematic, and customized guidance according to each activity will be needed, highlighting how rhythmic experiences could be further explored.

#### Raising Bodily Awareness Through Real-Time Multisensory Feedback

For participants, audio feedback served more than a technical feature; it is a medium that cultivates and maintains embodied awareness. The sound guidance raises present-moment consciousness, drawing participants into a close connection with their rhythmic movements. P3 explained the bridge between technological instruction and bodily understanding:

I focused on the beat and the sound. And there it (the synchronization) was much better.

P3, female, 70 years

This audio-mediated awareness helps to orient the walking experience into a contemplative practice. The synchronization between sound, step, and heart rate interweaves into a dynamic movement, where participants learned to listen and resonate not just with their ears but with their entire moving body.

Notably, this dynamic movement was fragile, easily disrupted by environmental distractions or cognitive overload, as mentioned in the Disrupted Natural Flow section, revealing how audio-guided synchronization demanded sustained and focused presence. To help with audio-guided synchronization, the integration of visual feedback created a richer source of engagement and accomplishment. Visual cues, including the changing numbers and colors, functioned as an immediate representation of participants’ efforts, making abstract concepts of “being in sync” intuitively observable. With the interactive app interfaces and sounds, the compound feedback system gains a more meaningful and motivating guiding quality.

The device’s capacity to provide a real-time compound feedback functioned as an extension of participants’ bodily awareness during exercise. P13’s experience demonstrated how RTF could recalibrate habitual movement patterns: “*Otherwise, I would walk too fast all the time*.” This suggests that the device helps develop enhanced self-awareness, helping participants recognize and modify unconscious movement tendencies. For P1, the discovery of elevated heart rate during brief exercise periods created a sudden awareness of her body’s responses that had previously remained hidden. This demonstrates how technological feedback could generate moments of surprise, revealing aspects that might otherwise remain unconscious. The real-time nature of the feedback opened up opportunities for immediate embodied learning, where wearers integrated technological information into the lived experience.

### Wearables With RTF: Interpretation of Data

#### Overview

Participants’ relationships with health data revealed intricacy between curiosity, confusion, and desire for meaning-making. The pulse data held significance as a direct representation of their most intimate bodily rhythm by making their heartbeat visible and quantifiable. This transformation of internal and felt experience into external and observable data created both fascination and interpretive challenges.

#### Relational Meaning-Making of Health Data

The participants struggle to understand how the data represent their health. Participants thus expressed a need for consultation and interpretation of data. P18’s reflection captured this essential need:

It’s fun (to get the data). But with the data, I would like to have a discussion (with others or professionals), so I can also understand the data.

P18, female, 67 years

This desire revealed that health data was not merely informational but deeply relational for these participants. The meaning of their physiological information emerged not in isolation but through conversation, interpretation, and contextualization within their broader life experiences.

The absence of data interpretation left participants with pieces of embodied information that lacked coherent meaning or actionable significance. P6’s confusion, “*I do not understand why I get this information*,” also highlighted how health data without interpretive support could create disorientation rather than empowerment. This suggests that meaningful engagement with data requires not just data provision but spaces for sense-making, where technological information becomes integrated into participants’ knowledge of their own well-being.

#### Technological Barriers Hindering PA Enhancement

Participants’ encounters with technical challenges revealed how technological complexity could fracture the intended engagement. P7’s struggle with volume settings exemplified how technical barriers could create frustration. Also, P11’s frustration revealed how technological barriers could create alienation from one’s own embodied data, transforming what should be empowering information into a source of confusion and inadequacy.

I could not find information, like my heart rate, since I didn’t know where to look for it.

P11, female, 70 years

These moments of technological breakdown highlighted the fragility of the wearer-device relationship and the importance of seamless integration for meaningful embodied engagement, so that the technological exclusion will not alienate the participants from the device’s capabilities due to the technical limitations. This tension highlights the mismatch between technological promise for enhanced bodily awareness and the reality of technical complexity for wearable devices.

### Wearables With RTF: Temporal Trajectories of Device Engagement

#### Overview

During the 3-week study period, the 17 participants (P1-P18, excluding P8) showed highly variable engagement with the device. While the recommended usage was 30 minutes per session, 3 times weekly (270 minutes total), actual usage time ranged from 51% to over 700% among the participants. P2 used the device least (137 minutes, 51% of recommended), while P9 showed the highest engagement (1905 minutes, 706% of recommended). On average, participants used the device for 720 minutes, nearly 3 times the recommended amount (267%).

We grouped participants into 3 categories based on total usage time—limited engagement (≤150% of recommendation) included 6 participants (P2, P3, P5, P11, P13, and P14), moderate engagement 150%-300% of recommendation) comprised the majority with 7 participants (P1, P4, P10, P12, P15, P16, and P18), and high engagement (≥300% of recommendation) included 4 participants (P6, P7, P9, and P17). The threshold of 150% and 300% was chosen based on the empirical distribution of the data and interpretability considerations. When we examined the actual usage patterns, they emerged as a natural break point that meaningfully separated participants with different engagement levels with the device.

Session patterns also varied considerably, with some participants preferring fewer but longer sessions (eg, P4 and P9 averaged over 60 minutes per session) while others engaged more frequently with shorter durations (eg, P15 and P16). Detailed engagement data and participant groupings are provided in Multimedia Appendix 5.

The 3 groups of device usage time became a lens through which different dimensions of the lived experience unfolded. Wearer-device relationships evolved with usage time. Limited engagement often brought frustration, moderate use led to reflection, and extended engagement fostered mastery and integration. Over time, some users shifted from resistance to routine, showing how long-term interaction could transform technology from a burden into a meaningful part of PA.

#### Disruption and Emerging Connection in Limited Engagement

For participants who engaged themselves for shorter durations (≤148% of the recommended 270 minutes, ie, ≤400 minutes), the experience was characterized by a critical awareness of bodily disruption and cognitive demand. P11 (97%) described her encounter with the device as requiring her to alter her way of exercising:

I am a fast walker, so walking too fast got uncomfortable (to synchronize with my heart rate). It required too much effort and focus to follow the exact goals.

P11, female, 70 years

This disruption of habitual movement patterns created a tension between her natural bodily rhythm and the technologically mediated pace. P13 (112%) experienced a similar disruption of her natural walking cadence, describing the necessity to walk more slowly as counterintuitive:

My heartbeat often was between 70 and 80, and that is really slow to walk. But it was hard to walk that slowly... You have got to go really slowly to be in time, in sync.

P13, female, 78 years

Despite this challenge, she found meaning in the device’s auditory feedback, which became a source of guidance and positive effect:

It is helpful when (the sounds) tell you when you are not in sync... There is a happy telephone sound when you are in sync.

P13, female, 78 years

P3 (132%), despite her limited usage time, experienced a profound transformation in her relationship with the device over time. Her lived experience evolved from initial struggle to proficiency:

The first week, I was 69% synced. The following week, I achieved 95% sync, and in the last week, I reached 97% sync.

P3, female, 70 years

This progression revealed how even limited temporal engagement could yield significant shifts in competence and connection with the technology.

#### Awareness and Growth in Moderate Engagement

Among participants who used the device between 148% and 333% of the recommended dosage (approximately 400-900 minutes), no particular positive or negative feelings were articulated toward the device. In this group, participants expressed a neutral observational attitude toward their synchronization performance. Interestingly, their awareness of the synchronization fluctuations during each session sometimes raises curiosity for them to reflect on their embodied experience. For example, P18 (156%) was fascinated by the dynamic, ever-changing nature of synchronization:

That is interesting to see how it fluctuates all the time... it really went, from like 61 (to) 68 and then it was 64 and then it went up to 78 and it was really up and down.

P18, female, 67 years

This temporal awareness revealed how moderate engagement fostered a contemplative viewpoint toward her own bodily processes.

Throughout the entire study period, P12 (272%) also experienced a changing synchronization rate, marking a journey of progression. His extended engagement with the device brought joy in improvement and a sense of achievement for him:

In the beginning, this was a hard day. Only 21%... but now this is 90%! Perfect.

P12, male, 72 years

Extended usage thus for P12 became a narrative of personal growth and technological competence.

### From Discipline to Integration in Extended Engagement

All participants who engaged with the device for extended periods (≥333% of the recommended dosage, ie, over 900 minutes approximately) developed strategies and demonstrated discipline when interacting with it, creating a nearly self-training process.

P16 (348%) expresses that she put in intense focus, which demanded a withdrawal from environmental engagement:

You must be so focused, extremely focused. If you see a nice sky or some flowers, you are not in focus again... You need to always concentrate on this device instead of this environment.

P16, female, 66 years

Her experience revealed the demanding nature of long-term synchronization, requiring a narrowing of perceptual attention.

P7 (567%) experienced the longest engagement with the device and developed a profoundly different relationship with the technology. For him, extended usage transformed the device from a demanding technological interface into a meditative companion:

In the end, I found that sound pleasant. It calmed me down... It reminds me of meditation... I did not have to think about anything.

P7, male, 76 years

His extended temporal engagement allowed for the emergence of a peaceful relationship with a familiar, internalized synchronization process.

## Discussion

### Principal Findings

In this study, we have explored the lived experiences of physically active older adults in using wearables for rhythmic activity, with a specific attention to RTF as an interaction modality. The study explored older adults’ previous use of wearables to track their PA, adherence with WRF during rhythmic activity, and the interactive features of WRF in enhancing the synchronization between step rate and heart rate. The study identified four main themes characterizing older adults’ lived experiences with wearables that are (1) use of wearable technologies without RTF in daily life, (2) embodied rhythmic negotiation with RTF, (3) interpretation of health data with RTF, and (4) temporal trajectories of device engagement with RTF.

While previous studies on wearables with older adults have recognized diverse motivations for adoption [11,12,33-35], this study reveals the specific mechanisms underlying this diversity in the context of real-time rhythmic guidance. Rather than treating heterogeneity as a static characteristic, we demonstrate how varied engagement patterns emerge through the dynamic intersection of personal values, embodied experiences, and technological demands. This study’s contribution extends beyond documenting diversity to explaining how older adults’ relationships with wearable technology are fundamentally shaped by their commitment to preserving authentic bodily experiences, their reliance on social networks for technology validation, and their need for contextually adaptive feedback systems that respect individual rhythmic patterns.

The study shows that the discontinuous use of wearables, accompanied by self-reported challenges in the device-guided synchronization, can be partly explained by the mismatch between technological capabilities and older adults’ inherent values and aspirations in later life. Factors such as comparison, competition, and goal setting that are often inherently built into commercial wearables [62] are less important for this demographic, for whom it is often more important to maintain the existing PA level through inner self-motivation. Simultaneously, device-guided synchronization requires older adults to change their natural walking rhythm and pace, which may result in a sense of discomfort and frustration. These age-specific requirements for achieving device-guided synchronization have largely remained unexplored in previous studies [30,58]. To overcome these challenges, the study proposes that interaction modalities that guide users to increase their bodily awareness may help older adults to find the right walking pace authentically.

The study addresses that older adults’ desire to use wearable devices stems mostly from their health-related goals and preferences, rather than their need for constant digital monitoring. These personal goals and preferences may show high variance in later life, which can partly explain the heterogeneity in driving factors to use wearables. Importantly, the findings reveal more than simple disuse but an intentional distancing from technology. This distancing aligns with research showing that older adults may abandon wearables when they are framed as assistive technologies that compensate for age-related losses, leading to negative user images [36]. Moreover, wearables can be perceived as motivating or condescending depending on how freely older adults are able to make autonomous choices about PA [34]. As a result, technology adoption among older adults involves complex value-based decision-making processes that prioritize the preservation of unmediated bodily experience over external validation.

Individuals’ readiness to accept wearable technology varies depending on their interests, experiences, and sociocultural background [32,63]. This study indicates that personal goals, often focused on the enjoyment of PA, were stated as the primary motivator to use wearables. These personal goals may sometimes contradict the device-related features that emphasize self-monitoring or goal setting in PA [34], which may partly explain the low or limited use of wearables in PA among older adults.

Previous research has explored the use of wearables by focusing on older adults’ motivations and driving factors at the individual level or from the device-centric perspective [11,33,35]. The study adds to this line of research by demonstrating the social aspects and motivations in the use of wearables for PA. The results demonstrate that technology adoption emerged not as an individual consumer choice, but as a socially embedded practice mediated through relationships, community connections, and shared experiences. This relational orientation, evidenced through participants’ learning via community seminars, family introductions, and the emotional significance attached to devices, challenges dominant narratives that frame wearable technology use as primarily self-directed and autonomous. This finding supports previous research indicating that formal and informal support and guidance in accessing and continuing device use are connected to long-term adoption [37]. The relational dimension extends beyond initial adoption to ongoing meaning-making, where device data interpretation requires dialogical engagement with professionals or trusted others. The combination of individual motivation, ease-of-use, and perception of added value ultimately determines whether devices will be integrated into users’ lives [11,33]. This shows that the role of family members or friends, also known as “warm experts” [17], remains significant in the uptake of wearables. Consequently, social support in the uptake of wearables, which is often unequally distributed in later life [64], could further diversify the heterogeneous use of wearables in PA.

The negative narratives we observed serve as a vital reminder that technology and health data can alter participants’ relationships with their bodies, shifting their view from an intuitive and instinctive feeling to one of external judgment and evaluation. The fact that decreased activity levels led to stress, rather than simply providing information or motivation, suggests that when technology introduces an evaluative aspect to PA, it might be emotionally challenging if not carefully designed. This emotional dimension is critical, as research demonstrates that long-term attachment with wearables requires devices to elicit positive emotional responses among users [40], and that emotional, sensory, and social influences significantly impact user engagement with wearables [13,38,39]. A long-term, motivating engagement with wearables also requires participants to develop strategies for managing the emotional impact, involving selective engagement and conscious boundary-setting around metrics. Fostering personalized approaches that preserve their sense of agency will be the key to selectively engaging with technologies that align with their values and needs.

### Lived Experiences of Wearables With RTF

The study reveals the significant age-related variations in user experience and adaptation to the wearable with RTF. Central to these findings is the recognition that rhythmic synchronization is deeply contextual, shaped by the interplay between bodily capacity, environmental demands, and technological requirements. The results demonstrate how synchronization challenges were not uniform technical difficulties but varied significantly based on environmental factors (flat terrain vs stairs and uneven surfaces), individual bodily characteristics (natural walking pace and heart rate variability), and temporal factors (time needed for adaptation). This aligns with research showing that through sound interaction, users can coordinate and stabilize movements with internal or external imposed rhythms [28], and that auditory feedback allows users to be observant of the environment and fits into more dynamic settings compared with visual and tactile guidance [27]. This contextual nature means that synchronization cannot be understood as a purely technical challenge, but rather as a complex negotiation between multiple intersecting factors that are dynamically configured in each exercising session.

Regarding synchronization challenges, older adults encountered difficulties synchronizing their steps to the audio feedback designed to align with their heartbeats. These challenges stemmed from a mismatch between the device’s requirements and the cognitive load, as well as from environmental constraints. Participants reported that synchronizing often required them to walk at an unnaturally slow pace, which conflicted with their habitual walking styles. This resulted in a perception that the device’s requirements were intrusive and disruptive to the natural flow of walking. Despite these challenges, older adults demonstrated an ability to improve their synchronization over time. This improvement, however, was not uniform across participants and appeared to depend on the individual’s personal exercise routines.

The study indicates that device-guided synchronization in rhythmic activity can be associated with older adults’ previous routines and rhythms, and thus connected to temporalities of PA. These insights suggest that while initial engagement with the device may be frustrating, persistence and strategic adjustments could enhance the experience of synchronization for some users. These adjustments, however, require “bodily knowledge and know-how” [65] to be able to adapt the device to already existing exercise routines. As Phoenix and Bell [66] highlight, disruptions in the natural movement rhythms may be associated with bodily discomfort and emotions, including symptoms associated with chronic health conditions connected to aging. These findings highlight that the device-guided synchronization, despite being a physiological mechanism between heart and gait cycles [30,58], is always connected to wider routines, rhythms, and temporalities in PA, as well as older adults’ previous exercise background and physical capabilities. In this study, the interconnectedness of synchronization challenges in relation to the environmental or activity-related factors was repeatedly pronounced in participants’ statements. This interconnectedness between synchronization and contextual and environmental factors may explain the reason for participants’ frequent statements of synchronization challenges, despite the fact that the device itself was considered relatively easy to use.

Overall, the device created both disruption and possibility, disrupting established movement patterns while opening space for new forms of bodily awareness and rhythmic experience. The challenge of synchronization revealed the depth of participants’ disconnection from their internal rhythms and their capacity for developing integration when given appropriate support and practice. This dual nature is inherent to RTF mechanisms, which provide haptic, auditory, or visual feedback simultaneously with user movement rather than after the activity has ended [2,3]. Notably, RTF has been identified as a preferred feature among older adults in wearable technologies [11]. In this sense, wearable devices with RTF function not merely as monitoring tools, but as mediators that can either fragment or enhance the relationship between consciousness and embodied movement, depending on how they are designed and implemented.

### Meaning-Making of Multisensory RTF

The meanings associated with the interactive features of WRFs extended beyond functional utility. The study addresses the potential of real-time audio feedback to help older adults overcome synchronization challenges and adapt to device-related functionalities over time.

First, RTF in auditory format can help older adults to reduce cognitive load in synchronization, which was considered to require an intense focus on finding and maintaining the right walking pace. Previous studies, respectively, have explored the auditory feedback in relation to increasing the enjoyment in walking activity, and showed that auditory cues could enhance the health benefit from walking among older adults through acting as a positive reinforcement to a rhythmic stepping pattern [67]. However, the degree of adherence to these feedback modalities depends strongly on individual characteristics, such as technological familiarity, cognitive capacity, and even sensory limitations. According to this study, older adults with more previous experience with technology tend to engage more easily with both visual and auditory feedback.

Second, the study shows that compound feedback, which combines visual and aural cues, improves synchronization and has a positive impact on the overall experience. Although its effectiveness may vary depending on individual needs and preferences, this strategy is especially helpful in fostering positive emotions like motivation and enjoyment. In order to accommodate a greater range of sensory abilities and preferences, wearable device design must be adaptive to an individual’s skills and capabilities. For example, older adults with hearing impairments may find auditory feedback less effective, while those with visual impairments may prefer larger visual cues.

Finally, temporal engagement with the device was not merely quantitative but fundamentally shaped the qualitative nature of participants’ lived experiences. Shorter engagements were characterized by disruption and effortful adaptation, while extended engagements allowed for the development of more integrated, contemplative, or mastery-oriented relationships with the technology. The duration of usage emerged as a critical factor in determining whether participants experienced the device as a disruptive intervention or as an integrated tool for embodied self-awareness, and potentially a natural component in PA. This temporal dimension is particularly significant given that adherence, defined as long-term user acceptance and engagement with devices, remains a persistent challenge in wearable technology research [4-6]. The individual factors of motivation, ease-of-use, and perceived added value that determine device integration into users’ lives [11,33] appear to require extended time periods for meaningful development. To sum up, technology adoption among older adults requires sufficient time for the development of new embodied competencies and the gradual integration of technological feedback into existing movement practices. The transformation from initial resistance or struggle to potential acceptance suggests that wearable technology design should accommodate extended adaptation periods rather than expecting immediate integration.

### Limitations and Future Research

The study gathered older adults with relatively high levels of PA and high satisfaction with their physical health. Therefore, their user experience with the device could potentially be influenced by their previous PA level. The participants used the device only for a 3-week period, and the long-term user engagement remained unexplored. Despite these limitations, this study has extended previous research by considering the lived experiences of older adults in device-guided rhythmic activity, with a specific focus on audio feedback during synchronization of step and heart rate. Future research should explore device-guided synchronization and adherence to RTF with a more diverse group of older adults. This would ensure that wearable technologies for self-monitoring can reach their expected health benefits for those older adults who are at risk of cardiovascular disease.

### Conclusions

This study reveals that older adults’ engagement with wearable devices for rhythmic activity is characterized by 4 fundamental dimensions that challenge conventional assumptions about technology adoption in later life. First, rather than simple disuse, older adults demonstrate intentional distancing from wearable technologies, actively resisting digital mediation to preserve authentic relationships with PA. Second, their engagement is fundamentally relational rather than individualistic, emerging through trusted social networks and requiring ongoing dialogical support for meaningful data interpretation. Third, the experience of rhythmic alignment is deeply contextual, shaped by dynamic negotiations between bodily capacity, environmental demands, and technological requirements that vary significantly across individuals and situations. Fourth, temporal trajectories of engagement reveal that meaningful relationships with wearable technology develop through extended interaction periods, with different engagement durations producing qualitatively distinct experiences ranging from disruption to integration.

These findings extend previous research on wearable technology use among older adults by moving beyond motivational factors to reveal the underlying experiential mechanisms that shape engagement patterns. The study demonstrates that device-guided synchronization creates both disruption and possibility, requiring extended temporal engagement for participants to develop integrated relationships with technological feedback. While RTF shows potential for enhancing bodily awareness, its effectiveness depends on designs that accommodate older adults’ commitment to embodied autonomy, their relational approach to technology validation, and the contextual nature of their rhythmic experiences. Future wearable technologies must therefore prioritize adaptive, socially embedded, and temporally sensitive design approaches that respect rather than override older adults’ established movement practices and values.

## Appendix

Multimedia Appendix 1 Questionnaire Design.

Multimedia Appendix 2 Pre-interview template.

Multimedia Appendix 3 Post-interview template.

Multimedia Appendix 4 Thematic analysis (themes, quotes, and commentary).

Multimedia Appendix 5 Device use among participants (n=18).

## Abbreviations

IPA: interpretative phenomenological analysis
PA: physical activity
RQ: research question
RTF: real-time feedback
WRF: wearable device with real-time feedback

## Data Availability

The datasets generated and analyzed during this study are available from the corresponding author on reasonable request.
