# Supplementary material for: Lived Experiences of Older Adults Using Wearables With Real-Time Feedback: Phenomenological Study
Source: JMIR Mhealth Uhealth. 2026 Apr 29;14:e71509. doi: 10.2196/71509 (PMC13173093; doi:10.2196/71509)
Supplement: Multimedia Appendix 1 [file mhealth_v14i1e71509_app1.docx]

## Appendix 1

**Questionnaire Design**

**A. Weekly Physical Activity Time: Multiple choice (time ranges from 0 minutes to 120+ minutes)**

- High-intensity physical activity *(such as running, sports, gymnastics)*

- Low-intensity physical activity *(such as walking, cycling, gardening)*

**B. Self-Perceived Health: 5-point Likert scale (1=very unsatisfactory to 5=very satisfactory)**

- Physical health

- Mental health

- Self-esteem

**C. Self-Efficacy: Numerical scale (0-100, where 0=can't handle it at all, 100=very confident I can handle it)**

- Confidence in maintaining regular exercise *(3+ times weekly)* across various challenging situations such as illness, stress, poor weather, family problems, etc.

**D. Quality of Life: Multiple choice (5 levels)**

- Mobility and walking ability *(from no problems to can't walk)*

- Self-care such as completing washing, dressing *(from no problems to can't wash/dress)*

- Daily activities such as work and household tasks *(from no problems to can't do)*

- Pain/discomfort levels *(from no pain to extreme pain)*

- Anxiety/depression *(from not anxious/depressed to extremely anxious/depressed)*

**E. Background Information:**

- Birth year: Open numerical answer *(year)*

- Gender: Multiple choice *(Man/Woman/Other/Prefer not to answer)*

- Marital status: Multiple choice *(Married/Unmarried/Cohabitation/Divorced/Other)*

- Children/grandchildren: Multiple choice with open input *(Yes with number specification/No)*

- Living situation: Multiple choice *(alone/with partner/with family/senior residence/other)*

- Additional comments: *Open-ended text*
