# Supplementary material for: Lived Experiences of Older Adults Using Wearables With Real-Time Feedback: Phenomenological Study
Source: JMIR Mhealth Uhealth. 2026 Apr 29;14:e71509. doi: 10.2196/71509 (PMC13173093; doi:10.2196/71509)
Supplement: Multimedia Appendix 2 [file mhealth_v14i1e71509_app2.docx]

## Appendix 2

**Pre-interview template**

**Welcoming**

Thank you for your time and for being part of this study. This interview aims to understand your physical activity habits and previous technology use. We will take this information into consideration when aiming to develop more inclusive and accessible wearable technologies for physical activity. We are interested in your experiences—both positive and negative—with physical activity and technology use. The interview will be confidential, and all data will be carefully anonymised. Your personal name or any other identifying details will not be published.

**1. Introductory Questions**

- Could you please tell us a little about your life situation, such as your age, occupation, and household type?

**2. Physical Activity – Habits and Motivation**

- Could you please describe a typical day in your life? Do you participate in any form of physical exercise or fitness activities? If yes, could you tell me about the type of exercises you do and how often you engage in them?
- How often do you exercise? What type of exercise?
- Have your physical activity levels remained the same during the past years? Or have you increased or decreased your physical activity recently?
- What motivates you to exercise?
- Do you ever feel that doing physical activity is challenging—or that it requires additional effort to start exercising? Do you feel day in and day out that physical activity can be a challenge in some way?
- How do you overcome these challenges?
- Do you like to train individually or in a group?
- We are also interested in your bodily experiences of doing physical activity. Can you describe how your body reacts to physical activity? Do you feel that your energy level increases, or do you experience some kind of pain after exercise? Or both? Does this sensory experience change during or after exercise?
- Does physical activity bring other benefits to your life? Do you feel it impacts your nutrition or quality of sleep? In what way? Do you have any examples of when there are mental or psychological benefits to exercise?

**3. Perceived Health and Well-Being**

- How do you experience your physical health at the moment?
- Do you have any physical health conditions that might affect your ability to do physical activity?
- How do you experience your (mental) well-being at the moment?
- Do you have any conditions or life situations that might affect your motivation to do physical activity?

**4. Use of Technologies for Physical Activity**

- In general, how comfortable are you with using new technology in your daily life? Are there any specific technologies or devices that you find particularly useful or challenging to use?
- How comfortable are you with using new technologies in general, such as smartphones, tablets, smartwatches, or computers?
- Do you use any technology for measuring your physical activity level?
- What kind of technology do you use?
- Have you ever tried any fitness application for tracking your physical activity? What kind of application?

***If the person has used any fitness application:***

- How long have you used the application? Do you use only one or several applications?
- What kind of information do you find useful in the fitness application?
- What motivates you to use the application?
- Does the application influence your physical activity level? In what way?
- Do you feel that the application helps you become more physically active? In what way?
- Do you think the application provides information that is beneficial for physical activity?
- Did you experience any difficulties using the fitness application?
- Did you experience any negative impacts of using the fitness application?

***If the person has not used any fitness application:***

- Is there any specific reason why you have not used any fitness application?
- Would you be interested in trying a fitness application? What kind?
- Do you believe that the application could help you become more physically active? Why/why not?
- What kind of information would you consider useful for enhancing your physical activity?

**5. Digital Skills**

- How would you evaluate your digital skill level? Do you perceive yourself to be an expert, beginner, or something in between?
- What kind of technology do you usually learn easily?
- Are there some technologies that are difficult for you to learn?
- Have you experienced any difficulties learning new technology?
- If you experience difficulties in using or learning new technology, how do you overcome them?

**6. Use of Wearables for Physical Activity**

- We are interested in your expectations for sensor technologies.
- Have you ever used any wearable device for physical activity?

***If the person has used a wearable device:***

- Could you share a specific situation when and where you used the wearable device?
- What was the main reason you started using a wearable device?
- What was your experience with the wearable device? Do you think it helped you in your physical activity?
- Did you notice any other benefits from using the device?
- Did you have any challenges, drawbacks, or limitations when using the device?
- Was it easy to start using the wearable device?
- Where (on which body part) did you wear the device? (waist, ear, neck, chest, head)
- Which one of these locations do you like the most/the least? Why?
- Did you receive any feedback (health-related information) from the wearable device?
- If so, what kind of feedback did you perceive as useful or less useful?
- Do you like the idea of receiving feedback from the device?
- What kind of feedback from the device would motivate you to do more exercise?
- Would you be able to imagine having a conversation with the wearable device?
- Did you experience any negative emotions when using the device? For example, needing to do more exercise without wanting to, or anxiety about doing too little exercise.
- Did you experience any negative bodily experiences when using the device? Such as pain, training too hard, or training without wanting to.

***If the person has not used a wearable device:***

- If you were to use a device, where would you prefer to wear it? (waist, ear, chest)
- What kind of information would you like to receive from the device (e.g., physical activity level, increase or decrease in activity, heart rate, other)?
- We would like to develop the device to be more interactive. Would you be comfortable using sound or voice to receive information about your physical activity?

**7. Physical Activity and Social Needs**

- Have you ever participated in group exercises or fitness classes? If yes, could you share an experience where you interacted with others while engaging in physical activities? Do you prefer group exercises or to exercise alone?
- Do you have friends who use wearable devices? Have they recommended them or complained about them to you? Does this affect your willingness to use wearable devices? Do you want to use the same devices as your friends?
- Have you ever recommended or assisted others in adopting wearable devices or similar technologies? If yes, could you share a specific instance and describe the outcome or impact of that recommendation?

**8. Final Thoughts**

- Is there anything else you would like to share about your experiences with wearable devices, or any other thoughts you have regarding their potential benefits or drawbacks for older adults?
