# Supplementary material for: Lived Experiences of Older Adults Using Wearables With Real-Time Feedback: Phenomenological Study
Source: JMIR Mhealth Uhealth. 2026 Apr 29;14:e71509. doi: 10.2196/71509 (PMC13173093; doi:10.2196/71509)
Supplement: Multimedia Appendix 3 [file mhealth_v14i1e71509_app3.docx]

## Appendix 3

**Post-interview template**

**Welcoming**

Thank you for taking the time to participate in this study. This interview focuses on your experiences using the WRF system for physical activity. Your insights will help us better understand how such technologies can support older adults in staying active and healthy. We are interested in hearing about both the positive and challenging aspects of your experience. Please note that your responses will remain confidential, and any identifying details will be fully anonymised. Your name or other personal information will not be included in any published results.

**1. Experience with the WRF**

- Can you share a specific occasion when you used WRF and how you experienced it?
- What was the main reason you started using a wearable device? Why did you begin using it?
- What was your experience with the wearable device? Do you think it helped you with your physical activity?
- Do you think the device helped you become more motivated? Why or why not?
- Have you noticed any other benefits of using the device?
- Did you face any challenges, disadvantages, or limitations when using the device?
- Was it easy to start using the wearable device?

**2. Feedback, Data, and Interaction**

- What do you think about wearing the device on your chest?
- Did you receive any feedback (health-related information) from the wearable device?
- If so, what type of feedback did you find useful or less useful?
- Do you like the idea of receiving feedback from the device?
- What type of feedback from the device would motivate you to exercise more?
- Could you imagine having a conversation with a wearable device?

**3. Emotional and Physical Responses**

- Did you experience any positive emotions when using the device? For example, joy from achieving goals.
- Did you experience any negative emotions when using the device? For example, feeling forced to exercise more than you wanted to, or anxiety about exercising too little.
- Did you experience any negative physical sensations while using the device? Such as pain, exercising too hard, or exercising without wanting to.

**4. System Usability and Future Use**

- Do you think you would want to use this device frequently? Why or why not?
- Do you think the system was unnecessarily complicated? Why or why not?
- Do you think the system was easy to use? Why or why not?
- Do you think you would need support from a technical person to use this system?
- Do you think the different features of the system were well integrated? Why or why not?
- Do you think there was too much inconsistency in the system? Why or why not?
- Do you think most people would be able to learn to use this system very quickly?
