# Supplementary material for: Lived Experiences of Older Adults Using Wearables With Real-Time Feedback: Phenomenological Study
Source: JMIR Mhealth Uhealth. 2026 Apr 29;14:e71509. doi: 10.2196/71509 (PMC13173093; doi:10.2196/71509)
Supplement: Multimedia Appendix 4 [file mhealth_v14i1e71509_app4.docx]

## Appendix 4

Table of Analysis Themes, Supporting Quotes, and Commentary

| **Superordinate Theme** | **Subordinate Theme** | **Participant Experience & Meaning-Making** | **Interpretative Commentary** |
| --- | --- | --- | --- |
| Use of Wearable Technologies without RTF in Daily Life | Embodied Autonomy and Resistance to Digital Mediation | *"I have a smartwatch, but I do not want to use it during physical activity. I keep it somewhere at home" (P2)*  *"I do not think it is that important to know how many steps... because I already feel it roughly" (P4)*  *"For me, that is why I like jogging and running. I do not want... It should be easy to train my body... just as easy as possible to move around" (P10)* | - actively resist technological intrusion  - defending authentic bodily relationships with PA |
|  | Contextual and Temporal Meaning-Making | *"(I found) a basic step-counter in the form of a phone-based application sufficient to track most basic physical activities" (P1)*  *"I used to have one (Garmin watch) many years ago when I was running. It was useful then, but now I do not use it anymore" (P13)*  *"I appreciate and I believe in technology. And I had tried several fitness apps previously" (P3)* | -active curation of technological complexity based on personal values rather than external recommendations,  - agency in technological engagement |
|  | Tensions or Ambivalent Emotional Responses | *"I got to know about my smart ring from a health engineer... she was the one who suggested this ring" (P17)*  *"I have a step calculator (on the phone). But I do not want to check it all the time. It is competitive and stressful... I think that is stressful" (P15)* | - technology legitimacy emerges through trusted others rather than individual choice.  - having stress when data conflicts with embodied knowing. |
| Wearables with RTF: Embodied Rhythmic Negotiation | Disrupted Natural Flow | *"I have realised that I have never followed my heart. I walk much faster" (P8, female, 71)*  *"I am a fast walker, so walking too fast got uncomfortable (to synchronise with my heart rate). It required too much effort and focus" (P11)*  *"My heartbeat often was between 70 and 80, and that is really slow to walk... You have got to go really slowly to be in time, in sync" (P13, female, 78)* | - disconnection from internal rhythms  -habitual movement patterns vs. technological demands.  - people feel estranged from their own physiological awareness |
|  | Learning and Guided Transformation | *"The first week, I was 69% synced. The following week, I achieved 95% sync, and in the last week, I reached 97% sync" (P3)*  *"I focused on the beat and the sound. And there it (the synchronisation) was much better" (P3)*  *"becoming like a dance" once synchronised (P3)* | -synchronisation becomes more meditative  - metaphorical view on movement  - achieve harmony through rhythmic learning. |
|  | Raising Bodily Awareness through Real-Time Multisensory Feedback | *"It is helpful when (the sounds) tell you when you are not in sync... There is a happy telephone sound when you are in sync" (P13)*  *"Otherwise, I would walk too fast all the time" (P13)*  *"I focused on the beat and the sound. And there it (the synchronisation) was much better" (P3)* | -audio-visual feedback creates new forms of embodied awareness  -revealing unconscious movement patterns  - immediate opportunities for self-correction or learning |
| Wearables with RTF: Interpretation of Data | Relational Meaning-Making of Health Data | *"It's fun (to get the data). But with the data, I would like to have a discussion (with others or professionals), so I can also understand the data" (P18)*  *"I do not understand why I get this information" (P6)* | - quantified information as incomplete without dialogical sense-making.  - need professional data interpreters’ help |
|  | Technological Barriers Hindering PA Enhancement | *"I could not find information, like my heart rate, since I didn't know where to look for it" (P11)*  *"I encountered technical frustrations with volume settings and interface navigation" (P7)* | - device didn’t help but became a source of inadequacy and confusion  - technological design can fracture rather than enhance their awareness. |
| Wearables with RTF: Temporal Trajectories of Device Engagement | Disruption and Emerging Connection in Limited Engagement | *"I am a fast walker, so walking too fast got uncomfortable... It required too much effort and focus to follow the exact goals" (P11)*  *"It is helpful when (the sounds) tell you when you are not in sync... There is a happy telephone sound when you are in sync" (P13)*  *"The first week, I was 69% synced... in the last week, I reached 97% sync" (P3)* | - short-term engagement shows a threshold  - participants prefer their familiar, habitual bodily patterns |
|  | Awareness and Growth in Moderate Engagement | *"That is interesting to see how it fluctuates all the time... it really went, from like 61 (to) 68 and then it was 64 and then it went up to 78" (P18)*  *"In the beginning, this was a hard day. Only 21%... but now this is 90%! Perfect." (P12)* | - witnessing some progress  - contemplative view on the device  - integrate technological competence with personal growth, creating meaning through the sense of achievement. |
|  | From Discipline to Integration in Extended Engagement | *"You must be so focused, extremely focused. If you see a nice sky or some flowers, you are not in focus again... You need to always concentrate on this device" (P16)*  *"In the end, I found that sound pleasant. It calmed me down... It reminds me of meditation... I did not have to think about anything" (P7)* | - Self-training mindset  - Internalised their interaction with the device's rhythmic guidance  - The device/cues become transparent to conscious awareness |
