# Supplementary material for: Lived Experiences of Older Adults Using Wearables With Real-Time Feedback: Phenomenological Study
Source: JMIR Mhealth Uhealth. 2026 Apr 29;14:e71509. doi: 10.2196/71509 (PMC13173093; doi:10.2196/71509)
Supplement: Multimedia Appendix 5 [file mhealth_v14i1e71509_app5.docx]

## Appendix 5

Table of device engagement among participants ( n=18)

| Participant ID | Total Time Used (min) | Adherence to Recommended Usage (%) | Number of Sessions Completed | Average Duration per Session (min) |
| --- | --- | --- | --- | --- |
| P1 | 571 | 211 | 18 | 32 |
| P2 | 137 | 51 | 6 | 23 |
| P3 | 357 | 132 | 9 | 40 |
| P4 | 684 | 253 | 11 | 62 |
| P5 | 278 | 103 | 8 | 35 |
| P6 | 909 | 337 | 17 | 53 |
| P7 | 1532 | 567 | 37 | 41 |
| P8 | missing | missing | missing | missing |
| P9 | 1905 | 706 | 30 | 64 |
| P10 | 580 | 215 | 16 | 36 |
| P11 | 262 | 97 | 11 | 24 |
| P12 | 735 | 272 | 14 | 53 |
| P13 | 302 | 112 | 9 | 34 |
| P14 | 351 | 130 | 9 | 39 |
| P15 | 598 | 221 | 20 | 30 |
| P16 | 939 | 348 | 36 | 26 |
| P17 | 680 | 252 | 15 | 45 |
| P18 | 420 | 156 | 10 | 42 |

Table of participants grouping (n=18)

| **Limited engagement (≤150% of Adherence )** | **Moderate engagement (150–300% of Adherence)** | **Extended engagement (≥300% of Adherence)** |
| --- | --- | --- |
| P2 (137 min, 51%) | P1 (571 min, 211%) | P6 (909 min, 337%) |
| P3 (357 min, 132%) | P4 (684 min, 253%) | P7 (1532 min, 567%) |
| P5 (278 min, 103%) | P10 (580 min, 215%) | P9 (1905 min, 706%) |
| P11 (262 min, 97%) | P12 (735 min, 272%) | P16 (939 min, 348%) |
| P13 (302 min, 112%) | P15 (598 min, 221%) |  |
| P14 (351 min, 130%) | P17 (680 min, 252%) |  |
|  | P18 (420 min, 156%) |  |
